# Adolescents leaving mental health or social care services: predictors of mental health and psychosocial outcomes one year later

**DOI:** 10.1186/s12913-015-0853-9

**Published:** 2015-05-02

**Authors:** Jessica Memarzia, Michelle C St Clair, Matt Owens, Ian M Goodyer, Valerie J Dunn

**Affiliations:** Developmental Lifecourse Research Group, Department of Psychiatry, University of Cambridge, Douglas House, 18b Trumpington Road, Cambridge, CB2 8AH England; NIHR CLAHRC East of England (EoE), Douglas House, 18b Trumpington Road, Cambridge, CB2 8AH England; Department of Psychology, University of Bath, Bath, BA2 7AY England; Mood Disorders Centre, Psychology, University of Exeter, Exeter, UK

**Keywords:** Adolescent, Service transitions, Looked-after children, CAMHS, Longitudinal

## Abstract

**Background:**

UK service structure necessitates a transition out of youth services at a time of increased risk for the development and onset of mental disorders. Little is currently known about the mental health and psychosocial outcomes of leaving services at this time. The aim of this study was to determine predictors of mental health and social adjustment in adolescents leaving mental health or social care services.

**Methods:**

A cohort (n = 53) of 17 year olds were interviewed and assessed when preparing to leave adolescent services and again 12 months later. Their mental health and psychosocial characteristics were compared to a same-age community sample group (n = 1074).

**Results:**

At discharge 34 (64%) met DSM IV criteria for a current psychiatric diagnosis and only 3 (6%) participants met operational criteria for successful outcomes at follow-up. Impairments in mental health, lack of employment, education or training and low preparedness were associated with poor outcomes.

**Conclusions:**

The findings suggest the current organisation of mental health and care services may not be fit for purpose and even unwittingly contribute to persistent mental illness and poor psychosocial outcomes. A redesign of services should consider a model where the timing of transition does not fall at the most hazardous time for young people, but is sufficiently flexible to allow young people to move on when they are personally, socially and psychologically most able to succeed. Assessment of a young person’s readiness to transition might also be useful. A youth focused service across the adolescent and early adult years may be better placed to avoid young people falling through the service gap created by poor transitional management.

## Background

Approximately half of all lifetime mental disorders emerge in the teenage years with 75% revealed before the age of 25 [1]. The risk of new onset and recurrence before the third decade is high [2]. In the UK, however, service structure currently necessitates a transition at age 18 and people with ongoing mental health or psychosocial problems transfer to adult services at a time of marked risk to ongoing psychosocial development and well-being. From the mental illness and social adjustment perspective this age cut off is an ‘artificial boundary’ ([3], s30) creating a system which is ‘weakest where it needs to be strongest’ with risks to ongoing treatment and care ([3], s30), and often leads to a ‘cliff-edge of lost support as young people with mental health needs reach the age of 18’ ([4], p26.). Inefficiencies and weaknesses in the current UK system have been highlighted and triggered renewed focus in exploring services for the 14–25 year age group which includes looking at cross-service approaches to better manage this period of incident risk for mental illness and personal adjustment into more independent adult living [4].

The current study followed two vulnerable groups of young people, each facing transition from a child and adolescent centred service by their 18th birthday: child and adolescent mental health services users (CAMHSu; facing discharge, transferral to GP care, AMHS or other services) and looked after children (LAC; moving from local authority care, usually to independent living). We undertook this 2-source study because vulnerable adolescents with a childhood history of emotional and behavioural difficulties and care concerns may attend either or both services and may arise from the same pool of vulnerable adolescents at the population level. Attendance at either is associated with poorer mental health and psychosocial outcomes in adult life than the population at large regardless of which service is responsible for their treatment and care [5-13]. Indeed adolescents attending social services show prevalence rates of psychiatric disorder akin to those attending CAMHS [14].

Within community samples of young people adolescent unemployment [15] and impairment in school functioning [2] have been highlighted as factors associated with poor mental health in early adulthood. Studies of adolescents leaving local authority care suggest the late teenage years are a critical time when their health needs go unmet [16,17], and increases in mental disorder in the year after leaving may increase. Good preparation for leaving child and adolescent services is associated with more successful transition into adulthood (17) whereas leaving care early is associated with consequent poor mental health and substance misuse [18]. For adolescents leaving CAMHS the majority of transitions are reported as poorly planned or implemented and poorly experienced by the user [19-21]. An ‘optimal transition’ incorporating good planning, joint working, information transfer and ensuring continuity of care across services is desirable but rare (<5%) [19-21]. These studies highlight the importance of management and professional factors that underlie a good care transfer process [19]. In contrast, we remain relatively unclear about the contribution of adolescent mental health per se to ensuring good transition outcomes and young adult adjustment. Indeed there is currently little evidence regarding which psychosocial factors at discharge predict successful transition once youth services have been left. Investigating a sample of vulnerable and hard–to-reach adolescents we determined whether their current mental health as well as education and employment status and preparedness to leave adolescent services predicted successful adjustment in the post discharge year. For the purposes of this study we define a ‘successful outcome’ as showing improvement in or no worsening of diagnoses, psychological distress, mental wellbeing, education or employment status, and perceptions of services. Remission is defined as the presence of no more than 1 psychiatric symptom and unimpaired for at least 6 weeks when re-assessed at 12 months.

We hypothesised first that the timing and nature of discharge from adolescent services would not be appropriately related to current mental health status or the patient being in remission. Second we hypothesised that the proportion of vulnerable youth with mental health problems at discharge will be similar across CAMHS or social services. Third we hypothesised that mental illness at follow up over 12 months would be enduring and associated with poor psychosocial outcomes.

## Methods

### Recruitment

Young people facing transitions in their care were recruited from two sources; child and adolescent mental health services (CAMHS from a single trust) and local authority care services (LAC, via two neighbouring local authorities). The mental health trust and the local authority services were from the Eastern region of the UK. All 17–18 year olds facing transition were deemed eligible for participation in this study. The research team was reliant on health and social care staff to identify and recruit subjects. An estimated 220 were in scope for the study and 59 (27%) were successfully recruited. Written informed consent was obtained from all participants. Most (n = 51, 96%) gave consent for researchers to examine their psychiatric or social care notes.

The study groups were compared to a community sample of adolescents, the ROOTS cohort [22], to compare characteristics against population norms for young people developing from adolescence to adulthood for this region of the UK. This is an epidemiologically derived community sample of adolescents who were recruited at 14 years and reassessed at 17–18 years (n = 1074, mean age 17.49 years). All comparisons of the transition group with the ROOTS cohort utilised data collected from ROOTS at the 17–18 year reassessment point.

### Measures

#### Interview assessments

The Structured Clinical Diagnostic Interview for DSM-IV-Research Version (SCID-RV) [23] was used to assess lifetime, recent and current diagnoses. The behaviour sections from the Kiddie-Schedule for Affective Disorders and Schizophrenia (K-SADS) [24] and the Psychotic-like Symptom Interview (PLIKSi) [25] were incorporated into the SCID-RV interview because it has been shown that these symptoms are often overlooked in mental state assessments of adolescents [23,24]. Psychiatric diagnoses were made with use of the SCID-RV and informed by clinical consensus during meetings with senior clinical staff.

Two sub-tests from the WASI (Wechsler Abbreviated Scale of Intelligence) [26], matrix reasoning and vocabulary, were used to estimate general intelligence.

At follow-up, the interviews incorporated questions assessing transition experience, which included whether participants had felt prepared for their transition, if they had any unexpected shocks, surprises or problems, whether their transition was stable and successful, and if they had understood what would happen and agreed with the decision.

#### Self-Report Questionnaires (SRQs)

A bespoke demographic questionnaire assessed residential, education, employment or training status and any criminal activity.

The General Health Questionnaire-12 (GHQ-12) [27] was used to measure subjective current psychological distress. Higher scores indicate more psychological distress. A threshold of 11/12 is associated with a threefold increase in the odds of a psychiatric diagnosis [28].

The Perceptions of Barriers to Service use (PBS) instrument was developed and assessed young peoples’ feelings and beliefs about using mental health and care services. It is composed of 32 statements with ratings of agreement (1 “strongly disagree” to 5 “strongly agree”, 3 “unsure”). The maximum score is 160 with higher scores indicating more perceived barriers to service use.

The Warwick Edinburgh Mental Wellbeing Scale (WEMWBS) [29] is a 14 item scale with a maximum score of 70 and higher scores indicating better mental wellbeing. Research literature indicates 50 as the normative level in a healthy adolescent population [29-31].

On the basis of advice from the adolescent service users involved in the planning of the study, SRQs were administered on laptop computers (with headphones for those with lower reading ability).

### Procedure

At baseline, all participants were facing transition from either CAMHS or local authority care services and moving to a primary care service, adult specialist services, or no further care (CAMHS), independence or Staying Put (LAC). Follow-up interviews were held 12 months after first assessment. At baseline, all SRQs (except the WEMWBS), the SCID-RV and PLIKSi were administered. At follow-up, all SRQs, and the SCID and PLIKSi (plus transition experience questions in the interview) were administered. Participants were offered a £25 thank you at each time point in recognition of their contribution to the study. The study was granted ethical approval by the Essex 2 Regional Ethics Committee (reference 10/H0302/17) and was carried out in line with the Good Clinical Practice (GCP) guidelines.

### Data analysis

Analyses were conducted using Stata 12 [32]. Measures of association and tests for effects over time on mental health outcomes were conducted using chi-square, t-tests, McNemar’s tests, logistic, linear and longitudinal linear regressions controlling for baseline measures. A robust sandwich estimator was used to accommodate both unequal variances and skewed distributions. Non-independence across time was accounted for within a random effects term. *P* values of <0.05 were considered significant and *p*s <0.1 are reported due to the limited statistical power to detect small and medium effects in the study. We also report effect sizes when possible (Cramer’s V, where 0.1 is small, 0.3 is medium and >0.5 is a large effect; Cohen’s D, where 0.2 is small, 0.5 is medium, and >0.8 is a large effect; or Cohen’s *f*^2^, where 0.02 is small, 0.15 is medium and 0.35 is a large effect).

The recruitment source (CAMHS or LAC), gender and IQ were included as covariates in all possible analyses. With factors predictive of outcomes, we assessed whether recruitment source as a covariate had a significant effect in the regression model, and then if so, we investigated effects in each recruitment source independently.

## Results

In nearly all analyses, the patterns of means or frequencies were similar between recruitment. Unless explicitly stated all of these analyses showed no significant differences between adolescents recruited from CAMHS or LA respectively. We fully report and decompose any significant effect of recruitment source.

### Sample characteristics

Of the 59 vulnerable adolescents who agreed to participate six withdrew leaving 53 (27 LAC and 26 CAMHS Care Leavers) with baseline assessments. Forty-five (85%) were re-interviewed after 12 months (mean time elapsed = 12.89 months), but of the eight who were not re-interviewed, SRQ data was collected for one and case notes provided details of diagnoses, service use and transitions for all eight.

Table 1 describes sample characteristics at entry into the study.

**Table 1:** **Sample characteristics at entry into the study, with comparison to the community sample**

	**Transition group (n = 53)**	**ROOTS (n = 1074)**	**Comparison**
Age: baseline	17.34 (0.45)	17.49 (0.34)	*t*(1125) = 3.08, *p* < 0.005
Sex: female (n, %)	31 (59%)	600 (56%)	*p* = 0.67
*Achieved 5/+ GCSEs A*-C	21 (46%)	1068 (86%)	*χ* ^2^ = 35.65, *p* < 0.0001, V = 0.422
*Left school before compulsory age	10 (19%)	-	-
*Arrested before baseline	21 (39%)	-	-
*IQ^a^	97.41 (15.84)	105.74 (16.42; n = 277)	*t*(328) = 3.4, *p* < 0.005
*Verbal IQ	96.71 (19.85)	107.59 (16.72; n = 277)	*t*(323) = 4.14, *p* < 0.001
Performance IQ	98.04 (15.58)	101.37 (17.61; n = 277)	*p* = 0.21
*GE	37 (70%)	1014 (97%)	*χ* ^2^ = 26.46, *p* < 0.0001, V = 0.36

^a^IQ was assessed in the ROOTS sample using the WISC and the performance IQ was assessed using the block reasoning task, whereas in the transition sample, performance IQ was assessed using the WASI matrix reasoning task.

*Local authority care leavers scored significantly worse on *these measures compared to CAMHS care leavers.

As expected the vulnerable adolescents showed poorer educational achievements, lower IQ and a smaller proportion in ‘gainful employment’ (or ‘GE’; a categorisation of those reporting to be in education, employment or training) compared to the ROOTS population sample. Lower IQ (in particular, verbal IQ), fewer educational achievements and a lower proportion in GE were more characteristic of those recruited from LAC than CAMHS.

### Mental state characteristics

Thirty-four (64%) of the 53 young people left services with one or more current clinical diagnoses. This prevalence amongst the service leavers was significantly higher than that reported in the community cohort (151, 14% ROOTS; *χ*^2^ = 52.54, *p* < 0.001, V = 0.51). As expected affective, anxiety and eating disorders were more common in the CAMHS leavers (*χ*^2^ = 13.55, *p* < 0.001, V = 0.52). In contrast, however, there was only a trend for substance/alcohol use, behaviour disorders and ADHD to be marginally more prevalent among LAC (*χ*^2^ = 3.44, *p* = 0.06, V = 0.26).

Furthermore, lifetime psychotic-like symptoms and non-suicidal self-injury were significantly more prevalent in those leaving services compared to the community sample (PLIKS: N = 23 (44%) in the vulnerable group v. N =156 (15%) in ROOTS (*χ*^2^ = 20.22, *p* < 0.001, V = 0.32); NSSI: N = 29 (55%) of vulnerable group v. N = 143 (11%) in ROOTS (*χ*^2^ = 43.78, *p* < 0.001, V = 0.48)).

Both lifetime PLIKS and recent NSSI were associated with increased likelihood of any psychiatric disorder when facing transition; controlling for gender, recruitment source and IQ (PLIKS: OR = 4.39, 95%CI (1.18, 16.32), *p* < 0.05; NSSI: OR = 8.35, 95%CI (1.42, 49.27), *p* < 0.05).

As expected, having a current diagnosis was associated with a significantly higher GHQ score (controlling for recruitment source, gender and IQ: *β* = 4.47, 95%CI (.16, 8.78), *p* < 0.05).

PBS scores were also significantly higher in those with a current diagnosis (controlling for recruitment source, gender and IQ: *β* = 12.27, 95%CI (3.52, 21.01), *p* < 0.01).

### Transferrals and discharge outcomes from adolescent services

In those recruited from LAC group, 18 (67%) moved to independent living. Eight (30%) opted to remain in stable existing foster placements on the ‘Staying Put’ scheme and one moved back to their biological family home. The Staying Put scheme at the time of the study was only available to young people in education or training and in a stable foster care placement, however recent changes to the Children and Families Act (which came into effect in April 2014) will require local authorities to support young people to stay in foster care until age 21 [33], and many local authorities across England have also recently pledged to provide support and advice until age 25 [34].

At baseline assessment none of the LAC leavers were being referred to adult mental health services despite over half having a current DSM disorder and a third with psychotic like symptoms and/or NSSI when assessed. However, 16 (61%) had been referred during their lifetime. Of these 7 (43%) had been non-compliant in engaging with psychological services. For the CAMHS leavers, 18 (82%) were discharged from services to GP care, three (14%) transferred to adult mental health services and one to child and adolescent substance abuse services.

### The transitional period between adolescent and adult service use

We categorised transitions as either continuing to receive (from a new service or remaining in foster care under the Staying Put scheme [LAC]) or losing specialist support (discharge to GP, move to independent living; although both had access to some support from either their GP or 16+ workers, respectively). Of those with current disorder at baseline 26 (84%) lost intensive support after leaving adolescent services. Furthermore, eight (42%) of those with no psychiatric diagnosis at baseline continued to receive support (*χ*^2^ = 4.13, *p* < 0.05, V = 0.29), all of whom were LAC leavers (seven carried on in foster care on the ‘Staying Put’ scheme, and one moved back to their biological family home). Recent experience of NSSI (*p* = 0.65; no difference in discharge type according to recent NSSI or none) and PLIKS (*p* = 0.08; just one of 14 with recent PLIKS transitioned to continued support) were found to be unrelated to receiving continued support after transition.

### Mental health at 12 month follow up

At the follow-up assessment clinical diagnoses were present in 19 (41%) of the vulnerable group. Six individuals, who had been well at discharge, had developed new mental illness onsets over the follow-up period (McNemar's = 0.09). The proportion of those with a diagnosis was still significantly greater in the vulnerable group compared to the ROOTS cohort (*χ*^2^ = 18.28, *p* < 0.001, V = 0.3). There were no significant differences in the patterns of emotional (internalising, *p* = 0.93) and behavioural (externalising, *p* = 0.29) disorders across groups by follow up compared to those reported at baseline (Table 2).

**Table 2:** **Change in gainful employment (GE) status, mental health and wellbeing and perceptions of service use over the transition period**

**Measure**	**Pre-transition (n = 53)**	**Post-transition (n = 46)** ^**a**^	**Comparison over time** ^**b**^
no GE/	16(30%)/	18(36%)/	*p* = 0.45
GE	37 (70%)	32 (64%)	
Current Diagnosis	34 (64%)	19 (41%)	OR = 11.34, 95%CI(1.49, 86.34), *p* < 0.05
PLIKS (past year)	14 (26%)	6 (11%)	OR = 3.53, 95%CI(1.01, 12.36), *p* < 0.05
NSSI (past 6 months)	14 (26%)	5 (11%)	OR = 8.33, 95%CI(1.06, 65.53), *p* = 0.05
GHQ	12.06 (6.4)	12.17 (6.98)	*p* = 0.94
PBS	83.83 (13.71)	80.41 (18.55)	*p* = 0.13
WEMWBS	-	46.96 (8.85)	N/A

^a^45 participants were re-interviewed post-transition, but SRQ data (GHQ, PBS, WEMWBS) and case notes of diagnostic status were gathered for 46. Case notes also provided GE status information for a further 4 participants not available for post-transition interview.

^b^All analyses presented here were adjusted for with covariates of gender, recruitment source and IQ. Unadjusted analyses showed no difference in significant results; findings were the same without covariates.

Both PLIKS and NSSI at the second assessment were associated with current diagnosis at follow-up (PLIKS: Fisher’s exact *p* < 0.05, V = 0.37; NSSI: Fisher’s exact *p* < 0.05, V = 0.35).

Compared to baseline follow-up GHQ scores did not improve (M = 12.17). As at baseline, higher GHQ scores were found in those with current diagnoses (controlling for recruitment source, gender and IQ: *β* = 6.59, 95%CI (1.8, 11.4), *p* < 0.01). However recruitment source as a covariate had a close to significant effect (*p* = 0.06) in this model. When explored in each recruitment source individually, only those leaving local authority care showed higher GHQ scores with a current psychiatric diagnosis (*β* = 10.13, 95%CI (4.26, 16.01), *p* < 0.01), whereas those leaving CAMHS did not show a significant effect (*p* = 0.47).

Higher PBS scores at follow up were associated with current diagnoses (controlling for recruitment source, gender and IQ: *β* = 16.39, 95%CI (5.07, 27.73), *p* < 0.01). Mean PBS scores did not significantly change over the transition period (*p* = 0.13).

Mental wellbeing scores (WEMWBS) at follow-up were significantly lower (M = 46.95; SD 8.85) than the comparison ROOTS cohort (n = 1036; M = 51.59; SD 7.39; *t*(1079) = 4.09, *p* < 0.001); and were also significantly worse in those with a current diagnosis (controlling for recruitment source, gender and IQ: *β* = −7.77, 95%CI (−13.65, −1.89), *p* = 0.01).

### Gainful employment (GE) status

Similar proportions of vulnerable adolescents were classified as not in GE at follow-up (18, 36%) as at baseline (16, 30%). Those classified as in GE (32, 64%) at follow up had lower GHQ scores (*β* = −6.43, 95%CI (−11.35, −1.51), *p* < 0.05), and higher WEMWBS scores (*β* = 6.54, 95%CI (.27, 12.81), *p* < 0.05) when controlling for gender, recruitment source and IQ. Initial exploration of whether current diagnosis was associated with GE, controlling for gender, recruitment source and IQ, was non-significant, however, gender which was significant in this model. When studying each gender separately, females in GE were more likely to have a current diagnosis (controlling for recruitment source and IQ: OR = 9.02, 95%CI (1.02, 80.26), *p* < 0.05).

**Figure 1:**
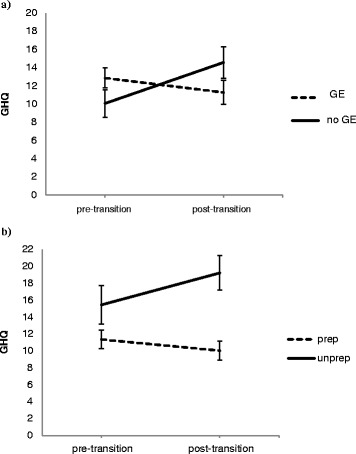
Mean GHQ score change across the transition period in those who **a)** were in gainful employment (GE) and those who were not in GE when facing transition and **b)** those who felt prepared and those who had not felt prepared for transition.

GHQ scores improved (i.e. reduced) over the follow up period in those who were in GE at discharge but increased over time in those who were not in GE (*β* = −6.36, 95% CI (−11.51, −1.2), *p* < 0.05; see Figure 1a).

### Preparedness for transition

Those at follow up who reported retrospectively not to have felt prepared for transition were more likely to have a current diagnosis (controlling for gender and group: OR = 9.09, 95%CI (1.51, 54.95), *p* < 0.05), even when accounting for baseline diagnosis (OR = 13.21, 95%CI (1.21, 144.37), *p* < 0.05), and were more likely to have developed a new disorder over the transition period (Fisher’s exact *p* <0.05, V = 0.42). Furthermore, they also showed a worsening of psychological distress (i.e. increased GHQ scores) over the transition period (*β* = −5.23, 95%CI (−10.07, −0.38), *p* < 0.05, *f*^2^ = 0.11) (see Figure 1b), and significantly higher GHQ scores at follow-up (*β* = 9.21, 95%CI (4.1, 14.31), *p* < 0.01). Preparedness was not related to baseline measures of psychological distress or psychiatric diagnosis (*p* = 0.13 & *p* = 0.52, respectively), and so was unlikely to be confounded by poorer mental health and distress when first assessed.

### Well-being and successful outcomes at 12 months

We computed an ‘optimal outcome standard’ based on 6 criteria assessed at 12 months follow up as follows: a) those who showed no worsening of GHQ scores (improved or constant) over the follow up period (n = 26); b) a GHQ score lower than the threshold of 11 post-transition (n = 19); c) no worsening of PBS score (improved or constant) (n = 24); d) a WEMWBS score at follow up equal to or higher than the normative value of 50 (n = 20); e) no current disorder at follow up (n = 27); and f) in gainful employment (n = 32). Although about 50% of participants met one or more criteria, only 3 (6%) participants could be defined as showing a successful outcome and none of these individuals had a current mental health disorder when leaving.

## Discussion

These vulnerable adolescents showed extensive and persistent psychosocial difficulties and psychiatric disorder after leaving youth services regardless of the organisation responsible for their care. The prevalence of psychiatric disorder was more than four times higher than observed in the local community. In addition, lifetime NSSI and psychotic-like symptoms were three to five times higher. In effect more than 80% of the participants showed persisting mental health, social or employment problems that were little changed over the 12-month follow-up period. In contrast, the ageist structure of mental health and social care services results in these vulnerable young people losing access to stable youth supports and entering an unfamiliar and highly variable system of care and service provision. Many young people felt poorly prepared despite well-documented policies and apparent provision for aiding the transition of each out of youth services.

These findings strongly indicate that the current age cut off of 17–18 years for delineation of services into adolescent and adult with entirely different staff, policies and procedures falls at a particularly hazardous period for mental health, social and adjustment problems for vulnerable youth [3]. The results suggest that the current service architecture is more likely to add to mental health burden, subsequent service use and increase cost to the UK taxpayer than to reduce undesirable outcomes. The recent discussion and change in legislation for an extension of service provision by local authorities for young people in care until age 25 is promising, as is the movement towards developing ‘youth services’ by mental health service providers modelling this in Birmingham, Ireland and Australia. However, it remains to be seen whether this provision for young people up to age 25 is feasible [35]. It may just create another arbitrary age cut-off criteria for transition, whereas our findings suggest that criteria ought to be informed by mental health and psychosocial factors of readiness.

The evidence further suggests that clinical remission or recovery by 12 months is influenced by a range of correlated factors at the time of discharge that include mental health status, gainful employment and/or maintaining education over the follow up period. This argues for much more integrated services and evaluations between mental health, social, education and employment services when formulating a transition plan.

The retrospective self-perception of not feeling prepared for transition also suggests a subjectivity factor in the path to recovery regardless of the aforementioned factors. Preparedness is increasingly being viewed as important, from studies of service users’ experiences [19,36] and is included in good practice guides for transition from child and adolescent services in many areas of health and care, in the UK including foster care services [37-40]. The development of ‘readiness checklists’ [41,42] for leaving services are currently being explored and their potential utility is supported by these findings. The senior author (VJD) is working with CAMHS leavers in three counties to co-produce a Transition Preparation Programme.

The low frequency of those with a ‘successful outcome’ in terms of mental health and functioning is comparable to the <5% experiencing ‘optimal transition’ as defined by service quality factors (proper joint planning, information transfer and continuity of care) reported by Singh et al. [19]; giving further importance to the reassessment of care service transitions policy and practice. Given that, in this report, psychosocial outcomes of young people leaving any services is substantially sub-optimal very serious consideration should be given to holistic improvement of transition policies and young adult services across adolescent service providers.

Overall we conclude that the age-based model of organisational transition at 17–18 years is not fit for purpose if the aim is to have a client-centred health and care system focused on the needs of the vulnerable young person; transition criteria, we suggest, should be based on mental health, psychosocial and preparedness factors rather than age cut-offs. Current service design appears to have arisen from historical top down influences from professionals and policy makers alike.

### Strengths and limitations

The small sample, resulting from the low recruitment rate, limits the results to a proof of principle investigation, particularly with our ‘subsample’ analyses where large confidence intervals suggest imprecise estimates of association. Further study with a larger sample is needed to validate the hypothesis-forming conclusions of this investigation. Our recruitment method, agreed with our NHS Trust and social care partners, required us to rely upon hard-pressed clinical and social care staff to identify and introduce the study to potential participants. This is likely to have contributed to our reduced sample as staff, working under considerable pressure, may have overlooked the introduction procedure amid the demands of a busy working day. It is also important to recognise possible sample bias due to staff selection of participants and loss to follow-up. Participant recall bias is a further possible limitation. However, we took a number of steps to minimise this: services used and episodes of illness were mapped chronologically on a timeline by the participant and researcher together, to provide context and improve accuracy; we applied high diagnostic thresholds and established significant impairment before diagnoses were given; information was corroborated in social care (LAC) and clinic (CAMHS) notes wherever possible.

## Conclusions

Adolescents discharged from mental health or care services may show a level of persistence of mental health difficulties and poor psychosocial adjustment at odds with the extant policies on recovery principles and client-centred service provision. Existing service architecture requiring a mandatory transition of care at 17 to 18 years of age may not be fit for purpose and may even contribute to enduring untreated mental illness and chronic poor functioning. Further study in a larger sample would be beneficial.
